# Temperature of a Dengue Rapid Diagnostic Test under Tropical Climatic Conditions: A Follow Up Study

**DOI:** 10.1371/journal.pone.0170359

**Published:** 2017-01-27

**Authors:** Onanong Sengvilaipaseuth, Koukeo Phommasone, Xavier de Lamballerie, Manivanh Vongsouvath, Ooyanong Phonemixay, Stuart D. Blacksell, Mayfong Mayxay, Sommay Keomany, Phoutthalavanh Souvannasing, Paul N. Newton, Audrey Dubot-Pérès

**Affiliations:** 1 Lao-Oxford-Mahosot Hospital-Wellcome Trust Research Unit (LOMWRU), Microbiology Laboratory, Mahosot Hospital, Vientiane, Lao PDR; 2 UMR "Emergence des Pathologies Virales" (EPV: Aix-Marseille university - IRD 190 - Inserm 1207 - EHESP), Marseille, France; 3 Institut hospitalo-universitaire Méditerranée infection, APHM Public Hospitals of Marseille, Marseille, France; 4 Centre for Tropical Medicine and Global Health, Nuffield Department of Clinical Medicine, University of Oxford, Churchill Hospital, Oxford, United Kingdom; 5 Mahidol-Oxford Tropical Medicine Research Unit, Faculty of Tropical Medicine, Mahidol University, Bangkok, Thailand; 6 Faculty of Postgraduate Studies, University of Health Sciences, Vientiane, Lao PDR; 7 Salavan Provincial Hospital, Salavan Provincial Health Department, Salavan Province, Laos; Universidade Nova de Lisboa Instituto de Higiene e Medicina Tropical, PORTUGAL

## Abstract

The Dengue Duo Rapid Diagnostic Test (SD Dengue RDT) has good specificity and sensitivity for dengue diagnosis in rural tropical areas. In a previous study, using four control sera, we demonstrated that that the diagnostic accuracy of these RDTs remains stable after long-term storage at high temperatures. We extended this study by testing sera from 119 febrile patients collected between July-November 2012 at Salavan Provincial Hospital (southern Laos) with RDTs stored for 6 months at 4°C, 35° and in a hut (miniature traditional house) at Lao ambient temperatures. The dengue NS1 antigen results from RDTs stored at 35°C and in the hut demonstrated 100% agreement with those stored at 4°C. However, lower positive percent agreements, with broad 95%CI, were observed for the tests: IgM, 60% (14.7–94.7) and 40% (5.3–85.3) for RDTs store at 35°C and in the hut, compared to those stored at 4°C, respectively. This study strenghtens the evidence of the robustness of the NS1 antigen detection RDT for the diagnosis of dengue after storage at tropical temperatures.

## Introduction

Imunochromatographic-based rapid diagnostic tests (RDTs) have multiple advantages, including providing results in less than 1 hour after sample collection, that can be performed at the patient’s bedside using a relative small volume of blood requiring limited technical skill to perform. This is especially relevant to low -resource countries where there is limited access to laboratory facilities. Over 2.5 billion people, nearly half of the world’s population, are now at risk of contracting dengue, and 70% of those live in southeast Asia and the western Pacific [1,2]. The SD Bioline Dengue Duo RDT (SD Dengue RDT, Standard Diagnostics, Alere, Waltham, Massachusetts, USA) has previously demonstrated greater than 80% sensitivity and specificity [3–7], for acute dengue diagnosis. The RDTs permit the concomitant detection of a dengue-specific antigen, NS1, on the left side cassette, and anti-dengue antibodies, IgM and IgG, on the right-side cassette. Therefore, this is a promising alternative for dengue diagnosis in Laos, and elsewhere in rural Asia [8–11]. However, the conditions of its use and storage in the field do not meet the standard of the laboratories where RDT evaluations are usually conducted. This is especially true during hot and rainy seasons when the storage temperature of the RDT storage may exceed the maximum temperature recommended by the manufacturer (30°C).

There have been few investigations of the effect of high temperatures on the accuracy of RDTs for dengue [12], malaria [13,14] and HIV [15]. Independent of the nature of the RDT, observations varied from no effect, to decrease in RDT accuracy, or to visual strip damage.

We published [16], a study on the effect of high storage temperature on the performance of SD Dengue RDT following exposure of RDT to high temperature (2 days at 60°C, 2 years at 35°C) or to field temperature in the long term (two years) and demonstrated that temperature did not affect the SD Dengue RDT diagnostic accuracy. However, a limitation of this study was that the tests were done using only four control sera (negative, NS1 positive, IgM positive and IgG positive). As a complementary follow on study, we present here the results using a series of febrile patients collected during 2012 at Salavan Provincial Hospital in Southern Laos using a range of RDT storage conditions.

## Materials and Methods

### Patients

One hundred twenty-four patients, admitted with fever (<8 days), without obvious cause, at Salavan Provincial Hospital (Southern Laos, 15.72 N and 106.42 E) from July to November 2012, were included. The patients gave written informed consent for this study of dengue epidemiology, that was approved by the Lao National Ethics Committee for Health Research and Oxford Tropical Research Ethics Committee. Venous blood was collected on admission and immediately centrifuged. Serum samples were kept at -20°C and then sent, within a month, on dry ice to Mahosot Hospital, Vientiane Capital and kept at -80°C until use.

### Description of RDT

The SD dengue RDT is an in-vitro immunochromatographic assay for the detection of dengue virus NS1 antigen and anti-dengue IgG/IgM antibodies in human serum, plasma or whole blood, from finger prick or venous blood. This test comprises a pair of test devices, a dengue NS1 antigen test on the left-side and a dengue IgG/IgM antibody test on the right-side. Each device contains a nitrocellulose membrane strip enclosed in a plastic cassette. The validity of the test is checked by the appearance of a control line on each strip. The test is easy to perform: 3 drops (using graduated dropper provided with the kit, ~100μl) and 10μl (using a graduated capillary provided with the kit) of sample are applied into the 2 small wells on the NS1 antigen and antibody cassettes, respectively. Four drops of diluent (provided with the kit) are then applied on the antibody cassette and the test results are read in 15 minutes. The cassettes are enclosed in individual hermetically sealed foil pouches containing a silica gel pounch that are packaged in cardboard boxes of 25 tests each. The manufacturer’s temperature range recommended for storage is from 1 to 30°C.

### RDT storage

Three hundred seventy-two SD Dengue Duo RDTs (Lot No 146011) were divided into 3 groups, each placed in different conditions: 4°C (4C_RDTs), 35°C (35C_RDTs) and in the hut (hut_RDTs—miniature traditional house as described [16]) for 6 months (from February to August 2015). 4°C was the temperature of reference, that corresponded to optimal storage conditions. The experimentation took place in Laos where temperature seasonally exceed 30°C and therefore 35°C was chosen to approximate typical tropical temperatures. The hut was used to mimic real condition in the field. The temperature in the hut was recorded every 90 minutes using electronic thermometers (Tinytag Ultra 2 data logger, temperature resolution of 0.01°C and accuracy of ~+/- 0.5°C, Gemini Data Loggers (UK) Ltd, Chichester, England) placed inside the RDT boxes. The mean minimum and maximum temperatures (95% CI, range) recorded in the hut during the study were 25.8°C (25.5–26.1°C, 18.7–29.8°C) and 35.7°C (35.1–36.3°C, 25.8–43.0°C), respectively.

### Performance of RDTs

Each serum was tested following manufacturer’s instructions in parallel on RDTs in each of the storage condtions. Two independent readers read all RDTs without conferring and blinded to the clinical details. Discrepancy between results from both readers (one reporting positive and the other one reporting negative for the same RDT) were considered as equivocal. A technical issue on the first day of the RDT testing required the exclusion of five samples due to application of insufficient diluent giving final total of 119 samples in the analysis.

### Statistical analysis

The result obtained with the RDT stored at elevated temperatures (35C_RDTs and hut_RDTs) were compared to the results obtained with the RDT stored in reference conditions (4C_RDTs). RDT results are presented in 2x2 tables and agreements (95% confidence intervals) were calculated as recommended by US FDA [17], using Stata v10 [18].

## Results

Comparisons of results obtained using RDTs stored in the different conditions are presented in Table 1 (results for individual patients are provided in supporting information S1 Table). Using the reference RDTs, stored at 4°C, 36 samples were positive for NS1 antigen, five for IgM and ten for IgG. Two samples gave equivocal results for IgG.

**Table 1:** Results of RDT stored at 35°C and in the hut in comparison to RDT stored at 4°C.

**NS1 results**		**Reference RDT stored at 4°C**			Total	Percentage agreements (95%CI)
		Pos	Eq	Neg		
**RDT stored at 35°C**	Pos	36	0	0	36	PPA: 100% (90.3–100)
	Eq	0	0	0	0	NPA: 100% (95.7–100)
	Neg	0	0	83	83	OPA: 100% (96.9–100)
Total		36	0	83	119	
**RDT stored in hut**	Pos	36	0	0	36	PPA: 100% (90.3–100)
	Eq	0	0	0	0	NPA: 100% (95.7–100)
	Neg	0	0	83	0	OPA: 100% (96.9–100)
Total		36	0	83	83	
**IgM results**		**Reference RDT stored at 4°C**			Total	Percentage agreements (95%CI)
		Pos	Eq	Neg		
**RDT stored at 35°C**	Pos	3	0	0	3	PPA: 60.0% (14.7–94.7)
	Eq	0	0	0	0	NPA: 100% (96.8–100)
	Neg	2	0	114	116	OPA: 98.3% (94.1–99.8)
Total		5	0	114	119	
**RDT stored in hut**	Pos	2	0	0	2	PPA: 40.0% (5.3–85.3)
	Eq	1	0	0	1	NPA: 100% (96.8–100)
	Neg	2	0	114	116	OPA: 97.5% (92.8–99.5)
Total		5	0	114	119	
**IgG results**		**Reference RDT stored at 4°C**			Total	Percentage agreements (95%CI)
		Pos	Eq	Neg		
**RDT stored at 35°C**	Pos	9	0	3	12	PPA: 90.0% (55.5–99.7)
	Eq	0	0	0	0	NPA: 97.2% (92.0–99.4)
	Neg	1	2	104	107	OPA: 95.0% (89.3–98.1)
Total		10	2	107	119	
**RDT stored in hut**	Pos	8	0	3	11	PPA: 80.0% (44.4–97.5)
	Eq	1	0	0	1	NPA: 97.2% (92.0–99.4)
	Neg	1	2	104	107	OPA: 94.1% (88.3–97.6)
Total		10	2	107	119	

Pos = Positive by both readers, Neg = Negative by both readers, Eq = Equivocal: discrepancy results between both readers. PPA: positive percent agreement ((positive by both RDT/positive by 4C_RDT)*100), NPA: negative percent agreement ((negative by both RDT/negative by 4C_RDT)*100), OPA: overall percent agreement (((positive by both RDT+negative by both RDT)/total)*100).

There was 100% agreement in NS1 results between 4C_RDTs, 35C_RDTs and hut_RDTs. There were no discrepancies between NS1 antigen results from both readers. Lower agreements, between 4C_RDTs and the 35C_RDTs and hut_RDTs, were observed for the antibody component. Overall agreements were good, from 94.1% to 98.3%. Percent agreements (95%CI) for dengue negative samples were good, 100% (96.8–100) for IgM and 97.2% (92.0–99.4) for IgG.

Among the five 4C_RDTs IgM positive samples, two gave negative results using RDTs stored at 35°C and in the hut and one gave an equivocal result using RDTs stored in the hut. Percentage agreements of the dengue-positive samples were low (60% for 35C_RDTs and 40% for hut_RDTs) and had wide 95% CI (14.7–94.7% for 35C_RDTs and 5.3–85.3% for hut_RDTs), due to the small number of positive samples. Among the ten 4C_RDTs IgG positive samples, one gave a negative result using 35C_RDTs and hut_RDTs, and one gave equivocal result when using the hut_RDT. Five 4C_RDTs IgG negative samples were IgG positive using 35C_RDTs (for three samples) or using hut_RDTs (for three samples). The two IgG equivocal samples were IgG negative using 35C_RDTs and hut_RDTs.

## Discussion

In this study 119 sera tested from suspected-dengue patients from a southern Lao rural provincial hospital were tested using RDTs stored at different temperatures for 6 months.

Perfect agreement was observed between the results obtained with these different RDTs for the NS1 antigen component. Thirty-five patients were NS1 positive regardless of the RDT storage conditions that demonstrated that the accuracy of this format was not affected by the storage of RDT at simulated tropical filed conditions.

Lowerer percent agreements were obtained with the antibody component. This was manifested by a reduction for the RDT sensivity for IgM detection when stored at higher temperatures, however, the magnitude of the issue cannot be accurately established due to the wide 95%CI due to limited number of positive patient samples.

NS1 antigen lines are always strong and easy to read. Therefore, a slight reduction in activity will not affect the line. However, with antibody component the lines are always much more difficult to read, and often quite feint, especially with low titer samples. So when there is a slight reduction in the activity due to the effect of heat, this is then manifests as lower reactivity (feinter lines which are more difficult to read) with low titer samples as they were always difficult to read. The samples we tested were acute samples with probably low antibody titers.

It is difficult to contextually compare the results presented here with previous studies that evaluated the SD Bioline dengue Duo RDT for dengue diagnosis [3–7] primarly due to methodological variations between the studies. Firstly, in this study we have used results from the 4°C RDTs as the reference comparator whereas other studies recruited dengue patients characterized using a comprehensive algorithm including clinical data, combinations of direct and indirect tests on paired sera to calculate overall sensitivity and specificity RDT result. In addition, in the current study the information on the days of fever prior to sample collection was not always available and it is recognized that this factor can influence the sensitivity of detection of NS1 and IgM [3,7]. Another source of RDT variation between studies and sites is that the loading of sample diluent, on antibody cassette, may not perfectly standardized since the volume of sample diluent could be different according to the way the bottle is squeezed producing small or big drops, as observed in Pal et al [5]. This could also explain the difference in performance we observed in this study between NS1 antigen and antibody components.

Therefore, our data suggest that antibody component of the SD Bioline dengue Duo RDT is affected by storage at increased temperature and would have a decreased sensitivity with samples of low antibody titer. Additionnal exepriments are needed on a larger panel of positive samples collected at various days of fever and of broad range of antibody titer. Our study streinghtens the robustness of the NS1 antigen detection for dengue diagnosis even after extended storage at tropical temperature.

## Supporting Information

S1 Table RDT results for each patient serum. (DOCX)
