# Correction: Temperature of a Dengue Rapid Diagnostic Test under Tropical Climatic Conditions: A Follow Up Study

**DOI:** 10.1371/journal.pone.0172658

**Published:** 2017-02-16

**Authors:** The PLOS ONE Staff

There is an error in the article title. The publisher apologizes for the error. The correct title is: Temperature Stability of a Dengue Rapid Diagnostic Test under Tropical Climatic Conditions: A Follow Up Study.

The correct citation is: Sengvilaipaseuth O, Phommasone K, de Lamballerie X, Vongsouvath M, Phonemixay O, Blacksell SD, et al. (2017) Temperature Stability of a Dengue Rapid Diagnostic Test under Tropical Climatic Conditions: A Follow Up Study. PLoS ONE 12(1): e0170359. doi:10.1371/journal.pone.0170359
